# Soft Contact Lens Engraving Characterization by Wavefront Holoscopy

**DOI:** 10.3390/s24113492

**Published:** 2024-05-28

**Authors:** Rosa Vila-Andrés, José J. Esteve-Taboada, Vicente Micó

**Affiliations:** Department of Optics and Optometry and Vision Sciences, Faculty of Physics, Universitat de València, 46100 Burjassot, Spain

**Keywords:** digital in-line holography, contact lenses, ophthalmic instrumentation, phase retrieval, digital image processing

## Abstract

Permanent engravings on contact lenses provide information about the manufacturing process and lens positioning when they are placed on the eye. The inspection of their morphological characteristics is important, since they can affect the user’s comfort and deposit adhesion. Therefore, an inverted wavefront holoscope (a lensless microscope based on Gabor’s principle of in-line digital holography) is explored for the characterization of the permanent marks of soft contact lenses. The device, based on an in-line transmission configuration, uses a partially coherent laser source to illuminate the soft contact lens placed in a cuvette filled with a saline solution for lens preservation. Holograms were recorded on a digital sensor and reconstructed by back propagation to the image plane based on the angular spectrum method. In addition, a phase-retrieval algorithm was used to enhance the quality of the recovered images. The instrument was experimentally validated through a calibration process in terms of spatial resolution and thickness estimation, showing values that perfectly agree with those that were theoretically expected. Finally, phase maps of different engravings for three commercial soft contact lenses were successfully reconstructed, validating the inverted wavefront holoscope as a potential instrument for the characterization of the permanent marks of soft contact lenses. To improve the final image quality of reconstructions, the geometry of lenses should be considered to avoid induced aberration effects.

## 1. Introduction

Contact lenses are one of the most popular ophthalmic solutions for refractive error compensation. Their geometry has evolved from the first rigid materials as PMMA to the introduction of softer hydrogel-based designs to the market in the 1970s [1,2,3]. Nowadays, several types of contact lenses are currently commercialized for clinical as well as for esthetical purposes. At the same time, they have become effective tools for the development of new biosensors capable of monitoring numerous biological parameters such as, among others, intraocular pressure and glucose levels, which act as biomarkers in different health problems [4,5,6,7].

Contact lenses usually include different types of permanent engravings, providing clinicians with information about their manufacturing process and the real position and orientation of the lenses once they are placed on the ocular surface [8,9]. Nevertheless, the nature of the lenses demands a proper geometry for the engravings, which are commonly performed with laser ablation of the lens’ material or over the manufacturing mould, and their depth values should be in the micron range. Moreover, a high level of transparency is needed for an acceptable esthetical aspect in addition to optimizing user comfort. Therefore, it is unsurprising that different techniques have been developed to ensure the best engraving functionalities with the minimum depth values [10,11,12,13]. These aspects (a high level of transparency and minimum depth values) might complicate observing permanent marks without a proper magnification, which is usually obtained in clinical in vivo performance by the utilization of slit lamps [9], the introduction of videokeratography techniques [14], or even a customized previous marking using different pigmentations to facilitate their detection in some research [15]. There are even some marks whose main purpose is to reveal the correct geometry to the user adaptation protocol for correct placement on the eye. The latter might also need to be slightly diffusive to become sufficiently visible.

The most common alternatives for the ex vivo observation of contact lens engravings and for the evaluation of their surfaces include the use of commercial interferometers [16,17,18,19,20,21,22,23,24,25,26] and microscopy techniques such as confocal, atomic-force, and scanning electron instruments [27,28,29]. Furthermore, Optical Coherence Tomography (OCT) [30,31] has supposed an efficient technique applied to the metrology of these lenses as well as, for instance, other technologies involving the introduction of fringe profilometry [32] and microtopography based on optical triangulation [33]. However, it is worth noting that the relief and transparency of contact lens marks make them weak-diffractive phase objects, accomplishing the Gabor requisites [34], thus being theoretically suitable for their characterization using digital in-line holography (DIH) in Gabor’s regime. Classically, this technique is based on the self-interference of an illumination beam passing through the sample over a photographic plate [34] which is substituted, in modern implementations, by a digital sensor [35,36,37]. Additionally, novel experiments have introduced several improvements in image capture and processing stages with approaches based on the introduction of tunable optical devices enhancing the hologram capture, focus distance, and magnifications [38,39,40,41]. More specifically, in this interferometric arrangement based on a classical Gabor in-line configuration, the reference beam directly comes as the non-diffracted light emanating from a partially coherent illumination source, and the object beam is created from the diffracted light coming from the illuminated sample, which is thus considered small in comparison with the reference beam (weak diffraction assumption). As a result of this interaction, the recorded hologram will provide access to the information of the inspected object, not only in terms of amplitude data, as in the case of conventional intensity imaging, but also in terms of phase values, which can be obtained from the propagation of the recorded hologram to an image plane using different numerical approaches [42,43].

In our case, the phase distribution is very important, because phase changes (Δφ) can be directly correlated with the optical path difference (OPD) coming from the inspected sample, which is proportional to its refractive index step (Δn), its thickness distribution (ΔL), or a combination of both parameters. This correlation is completed with the illumination wavelength in the form of ∆φ = 2π·Δn·ΔL/λ. In other words, the retrieved phase variations can be directly attributed with the associated OPD, allowing, for instance, for the numerical reconstruction of the sample’s geometrical shape (ΔL) for a known refractive index difference (Δn) between the surrounding medium and the sample or the opposite. Nevertheless, as a direct consequence of in-line configuration, an additional symmetrical image plane (the twin image one) will appear at the same distance from the sensor but at the opposite side. Therefore, the defocussed distribution provided by one of the images will overlap with the in-focus plane of the other image, causing a priori deterioration of the optical quality of the reconstructed image, a problem that can be solved by different image processing techniques [44,45,46,47,48].

Through the wavefront holoscope concept [49,50], this technology has been demonstrated to be efficient and cost-effective for the characterization of permanent marks in progressive addition lenses (PALs). These marks share some geometrical and functional similarities with the contact lens ones, but there are some important differences to consider. The first and most significant one comes from the curvature radius of soft contact lenses that needs to fit the ocular surface (anterior cornea surface) with a radius in the range of 8 mm, while the curvature radius in PALs depends on the lens power but is, at least, in the range of hundreds of millimetres. Second, the materials and the porous structure of soft contact lenses complicate their observation or manipulation in air (a medium where PALs can be easily inspected). For this reason, soft contact lenses are managed in cuvettes or special chambers filled with liquid solutions to avoid damaging (drying, shaping loss, etc.). This fact introduces a lower index step between the surrounding medium and the lenses when comparing them with the measuring conditions for the ophthalmic lenses, which can be directly observed in air. Thus, the OPD values are lower, resulting, as well, in lower phase values for the case of a similar geometrical depth of a mark. Third and closely related with the previous issue, there is another important difference related to the refractive index steps defined on both types of samples. For the case of the ophthalmic lens industry, it is common to engrave the marks on the PAL before covering the entire lens with different coatings (anti-reflective, UV, anti-glare, mirror, anti-fog, scratch-resistant, tinted, etc.), thus creating a sandwich of multilayers with different refractive index values over the mark, which gets completely embedded and may affect the value of the final OPD. Moreover, each manufacturer can apply a different multilayer sandwich depending on the PAL’s model and/or lens quality. Thus, it is difficult to precisely know the specific refractive index distribution for each inspected PAL to convert the phase (Δφ) into thickness information (ΔL). However, surface treatments are different in soft contact lenses, since they are aimed to be immersed in tears and the marks’ relief is still present. Therefore, knowing the value of the refractive index of the lens surface in contact with the media would be enough to calculate the OPD. In this case, cuvettes are typically filled with known liquids such as saline solutions, physiological serum, or distilled water, so thickness profiles can be easily obtained from the measured phase values. And finally, the dimensions of both refractive error-compensating systems are completely different. While PALs are typically in the range of 50 to 70 mm in diameter, contact lenses rarely exceed 14.5 mm (except for customized designs). This means that, whereas it is unreasonable to inspect the full optical surface in a PAL using digital in-line holographic arrangements, it is viable to do so in contact lenses by defining a specific experimental configuration which compensates its related aberrations.

In this paper, we present the experimental results of a profile characterization of permanent marks for soft contact lenses using a wavefront holoscope (w-holoscope) adapted to the geometry of contact lenses. We have named this new version i-wholoscope, which comes from inverted wavefront holoscope, since it is inverted in comparison with the initial w-holoscope device [49,50], as inverted microscopes also do in comparison with regular upright ones. Three commercially available soft contact lenses were inspected and their engraving profile characterized through the phase distribution retrieved by the customized i-wholoscope. Before that, a calibration of the device in terms of lateral resolution as well as depth accuracy is presented in Section 2 by virtue of well-known phase and amplitude test targets. The results and their analysis are described in Section 3 and Section 4, and the conclusions are presented in Section 5.

## 2. Materials and Methods

### 2.1. Inverted Wavefront Holoscope Assembly and Characteristics

**Figure 1:**
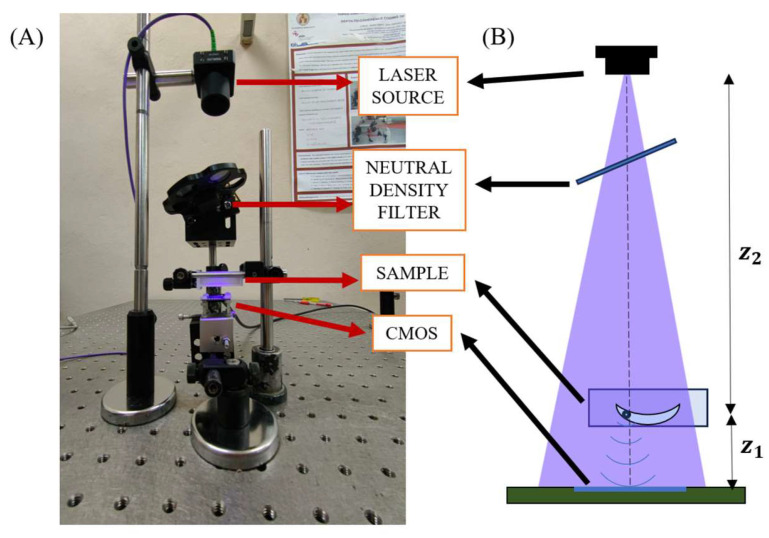
Experimental configuration for the i-wholoscope: picture at the lab (**A**) and a drawing (**B**) where the main components are clearly identified.

Figure 1 shows the assembled i-wholoscope as well as a scheme to easily identify its main components. The assembled i-wholoscope consists, firstly, of a divergent pigtailed diode laser source coming from a multiwavelength laser source (Blue Sky Research, SpectraTec 4 STEC4-405/450/532/635 nm, Milpitas, CA, USA), where the blue (450 nm wavelength) light was selected for the experiments. The diode is coupled to single mode fibre having a numerical aperture (NA) in the ~0.1 range, and the illumination source has driving, control, and stability electronics as well as temperature controllers to allow for high temporal uniformity in the emission. Moreover, the single mode fibre allows for a quasi-Gaussian shape in the illumination, thus providing a constant spatial distribution for the holographic recordings. Additionally, the intensity can be manually controlled by a neutral density filter, as shown in Figure 1. The sample, directly illuminated by the laser source, is located at a distance of z_2_ = 212 mm from the laser. It consists of a soft contact lens placed into a cuvette filled with a saline solution to conserve the lens morphology and replicate its regular conditions of use. The commercial contact lenses used in this experiment are the following ones: (i) Miru 1-Month Toric (Menicon^®^, Nagoya, Japan), (ii) Soflens Toric (Bausch & Lomb^®^, Vaughan, ON, Canada), and (iii) spherical Acuvue Oasys 1-Day (Johnson & Johnson^®^, Jacksonville, FL, USA). The vertical configuration of the optical setup enables a horizontal cuvette positioning, preventing gravity effects over the inspected lenses. Finally, a complementary metal-oxide semiconductor sensor (Basler daA2448-70 μm, 2448 × 2048 pixels, 2.74 μm pixel width, Ahrensburg, Germany), from now on named as CMOS, is located at a distance of z_1_ = 10 mm from the cuvette, thus obtaining a uniform illumination along the whole sensing area. This configuration provides a system magnification of approximately $M=\frac{z_{1}+z_{2}}{z_{2}}=1.05$ as well as a theoretical spatial resolution of $\rho_{s}$ = 5.23 µm (191 lp/mm), which comes from the sampling criteria introduced by the pixel width (∆p) of the CMOS $\left(\rho_{s}=2∆p/M\right)$. In this arrangement, the diffraction limit (ρ_d_ = λ/NA) can be computed from the numerical aperture values in the horizontal (NA_H_ = 0.32) and the vertical (NA_V_ = 0.27) directions, yielding, in a diffraction-limited resolution, values of ρ_dH_ = 1.41 µm and ρ_dV_ = 1.67 µm, respectively.

The image reconstruction process involves a back propagation of the recorded holograms to the image plane based on the angular spectrum method (ASM). Initially, the device was calibrated in terms of spatial resolution, with amplitude and phase USAF 1951 resolution test targets placed at a similar distance than the contact lenses are located, and the rest of the system was preserved. In addition, since our target lied on the mark characterization in shape and depth, the i-wholoscope was also quantitatively calibrated in terms of the recovered phase values, which can be directly correlated with the height of the marks. To allow for this, a phase target (Benchmark Technologies: https://benchmarktech.com/quantitativephasemicroscop/, accessed on 25 May 2024) composed by different phase details of known height was used, and the holograms of two focus star objects with a height of 330 and 278 nm were recorded (these values were measured and provided by the manufacturer). Their respective phase maps were obtained with back propagation to the real image plane, and the phase profile of each one was analysed, translating the obtained phase values to height values.

### 2.2. Digital Image Processing and Twin-Image Removal Process

**Figure 2:**
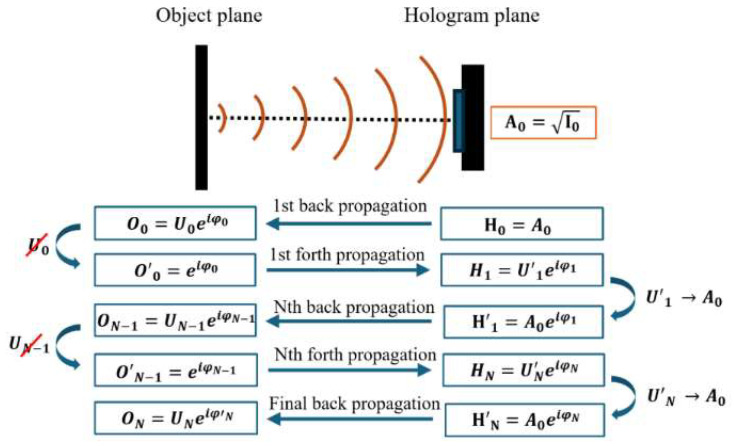
Diagram of the phase-retrieval algorithm based on the Gerchberg-Saxton method.

To improve the optical quality of the images, a phase-retrieval algorithm based on a classical Gerchberg–Saxton routine was used to remove twin-image disturbances. This algorithm, which is depicted in Figure 2, is based on an iterative routine which performs back and forth numerical propagations between the hologram and the real image planes using the ASM, conserving the initial amplitude of the recorded hologram but adding a tentative phase distribution coming from the real image plane, thus decreasing the contribution of the phase term related to the twin image in each iteration. This process starts with a first back propagation from the hologram to the real image plane. This stage assumes a non-existent initial phase distribution on the hologram plane, storing, on the computer memory, the information related to the initial recording amplitude (A_0_) of the hologram as the square root of the recorded intensity image and the propagation distance from the hologram to the real image plane. Once on the image plane, the amplitude values are cancelled, and it is only the remaining phase distribution information at the image plane that is forth propagated again to the hologram plane using the previously stored propagation distance. At this step, a new distribution is obtained at the hologram plane, for which its new amplitude is substituted by A_0_. This process enables one to obtain an enhanced hologram which will include the initial hologram amplitude but will be already complemented by an estimated phase distribution closer to the one provided by the real-image term. Finally, this new hologram can be back propagated again to the image plane using the initial propagation distance, where its amplitude value will be another time-cancelled one, thus following the same cycle for each iteration. This sequence can be repeated for a different number of iterations (N) depending on the initial quality of the engraved hologram, the diffractive nature of the inspected samples, and the expected results depending on the application.

The algorithm convergence and the induced effects of the number of iterations of the algorithm were studied by comparing the effect of a different N with the mean height of known-height objects from a calibration phase target and the standard deviation of its surrounding empty background. This can be understood as a metric for background inhomogeneities which ensures, on the one hand, that the theoretical thickness values are included in the measurement estimation ranges obtained with a different number of iterations and, on the other hand, that significant levels of high frequency noises are not added to the original background inhomogeneities calculated for direct back propagation before the phase-retrieval routine.

## 3. Results

### 3.1. Microscope Calibration

#### 3.1.1. Spatial Resolution Calibration

**Figure 3:**
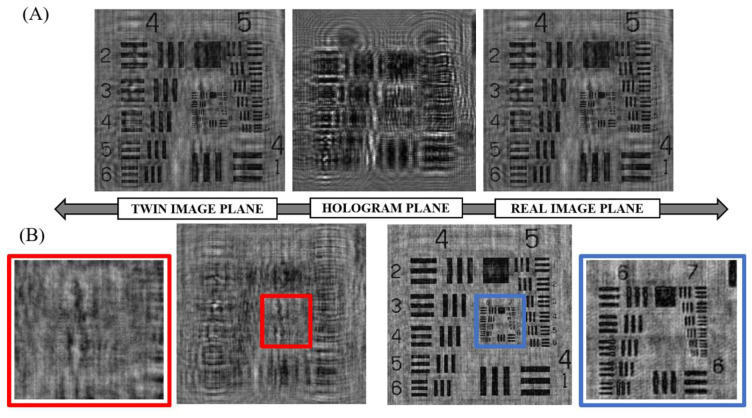
Back and forth propagated amplitude USAF target before (**A**) and after (**B**) removing the twin-image contribution using a phase-retrieval algorithm. The area containing the last resolved element is marked and zoomed with blue and red boxes in the real and twin images planes, respectively.

Figure 3 shows, in detail, the difference between the experimentally obtained intensities at the real and twin-image planes for an amplitude USAF 1951 target before and after the application of the phase-retrieval algorithm. In the case of the real image plane, it can be observed how the application of the iterative routine helps to improve the image quality without losing the spatial resolution, also obtaining a blurry image on the twin-image plane, whose contribution to the enhanced hologram was progressively removed cycle by cycle from the iterative procedure. As it can be observed, the smallest element resolved for the USAF amplitude target is the fifth element of the seventh group (G7-E5), corresponding to 203 lp/mm, in other words, a spatial resolution limit of 4.93 µm.

**Figure 4:**
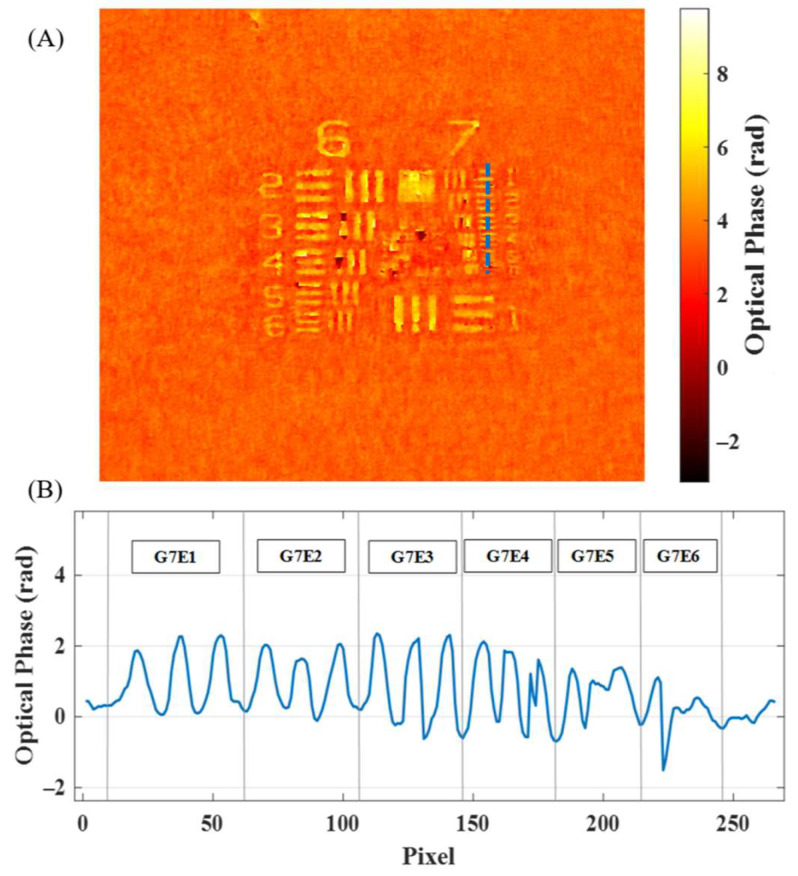
Focused USAF from the phase calibration target: (**A**) the 2D optical phase distribution outcoming from the phase-retrieval algorithm and (**B**) the plot along the vertical blue dotted line in (**A**) involving a profile of the different elements at the seventh group.

Additionally, Figure 4 presents the experimental results when considering the same digital methodology but using a phase USAF 1951 from the phase calibration target. Once again, the last resolved element is G7-E5, as shown by the plot along the blue dotted line included in Figure 4A,B.

#### 3.1.2. Thickness Calibration

**Figure 5:**
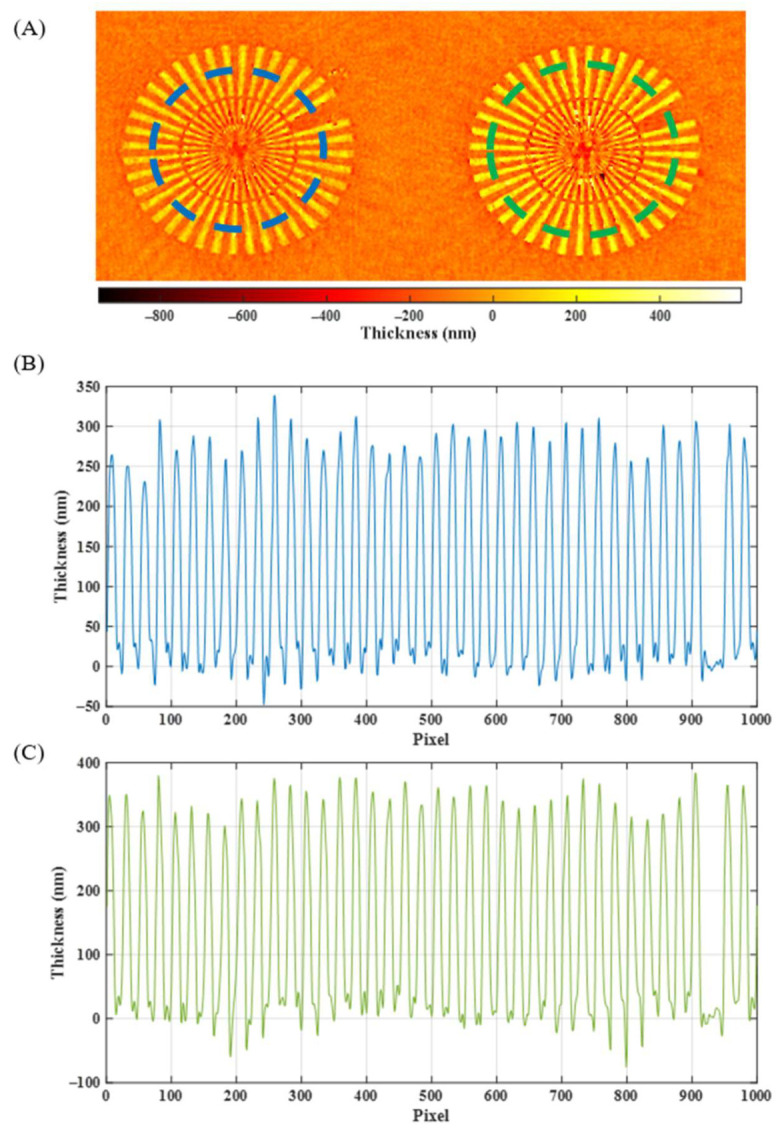
(**A**) Recovered phase maps of the recorded holograms for a 278 nm and a 330 nm focus star. (**B**) Experimental depth values obtained for a 278 nm theoretical focus star object along the blue dotted line. (**C**) Experimental depth values obtained for a 330 nm theoretical focus star object along the green dotted line.

To perform a calibration of the thickness measurement of the proposed system, a pair of focus star objects from the calibration phase target were previously measured. Their theoretical thickness values correspond with the manufacture’s estimated values of 330 nm and 278 nm. The obtained thickness results for both phase objects are represented in Figure 5, where the focused phase image of both objects (Figure 5A) as well as the obtained phase profiles for each one of them (Figure 5B,C) are depicted. Through the obtained experimental phase profiles, we computed the height values for both star targets, resulting in 352 ± 26 and 288 ± 27 nm, respectively, thus validating the i-wholoscope as a quantitative imaging platform for profile measurements.

**Figure 6:**
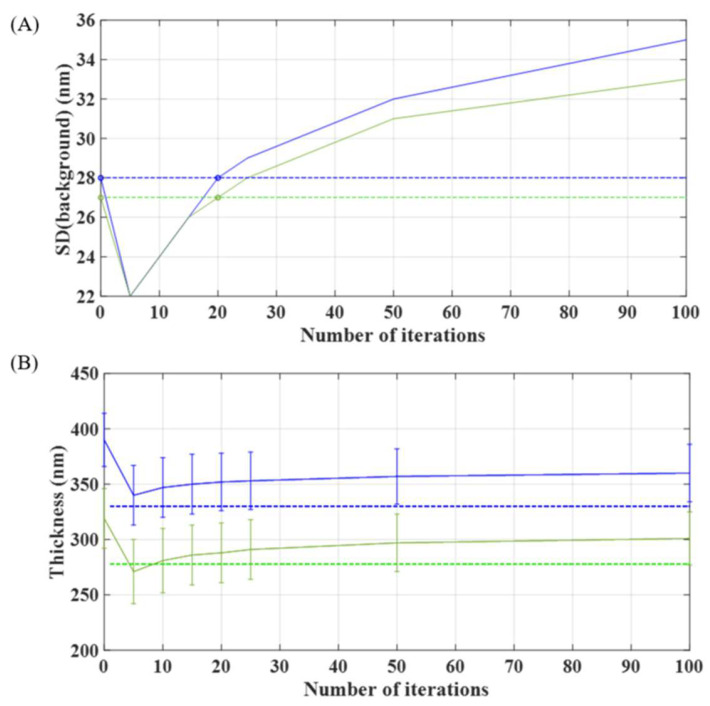
(**A**) Evolution of the standard deviation of the background of the calibration phase target as a function of the number of iterations of the phase-retrieval algorithm. (**B**) Evolution of estimated thickness values for both phase star targets as a function of the number of iterations of the phase-retrieval algorithm Values in green and blue correspond to the experimental results for the 278 nm and 330 nm theoretical focus star objects respectively.

These measured values were obtained for N = 20, which seems to be the proper number of iterations that can be applied to prevent the significant addition of high frequency noise (higher than the one existing in direct propagation, that is, N = 0) and to preserve an accurate height estimation (inside the error bar provided by the standard deviation—SD). The selection of this value is justified through the graphs included in Figure 6, where the number of iterations in the phase-retrieval algorithm is compared with the SD of the background in Figure 6A and with the obtained thickness results for the calibration targets in Figure 6B. For both cases, N = 20 seems to be a good, compromised value.

### 3.2. Characterization of Contact Lens Engravings

**Figure 7:**
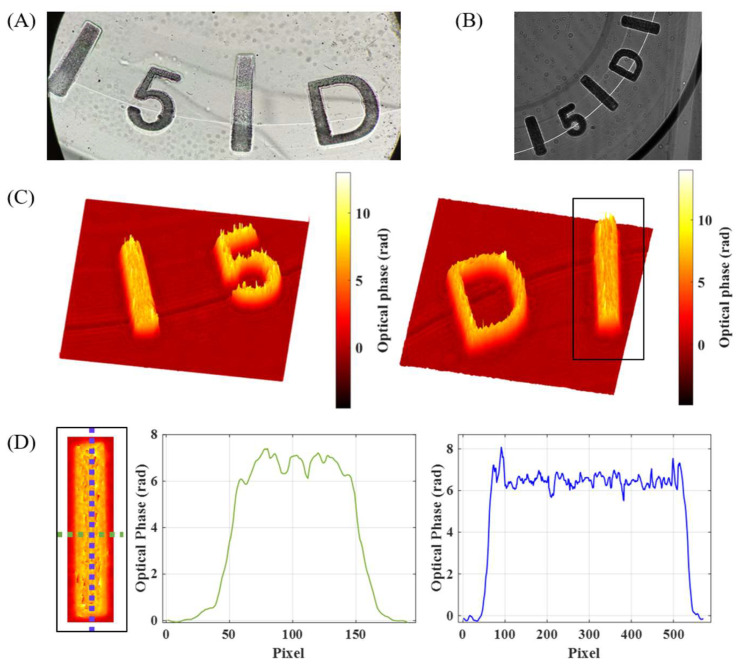
(**A**) Permanent marks of a Soflens contact lens observed with an optical Olympus BX-60 microscope. (**B**) Recorded hologram of the permanent marks of a Soflens contact lens. (**C**) Experimentally obtained phase maps of this contact lens. (**D**) Obtained phase profiles (horizontal/vertical ones in green/blue plots) from the mark indicated with a black box in (**C**).

**Figure 8:**
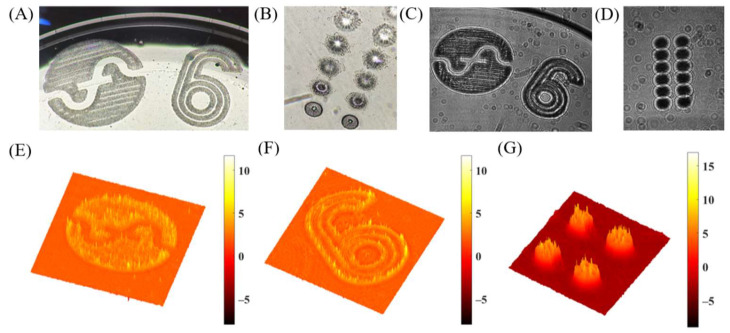
(**A**,**B**) Permanent marks of a Miru lens observed with an optical Olympus BX-60 microscope. (**C**,**D**) Recorded holograms of the permanent marks of a Miru lens. (**E**–**G**) Experimentally obtained phase maps for this contact lens.

**Figure 9:**
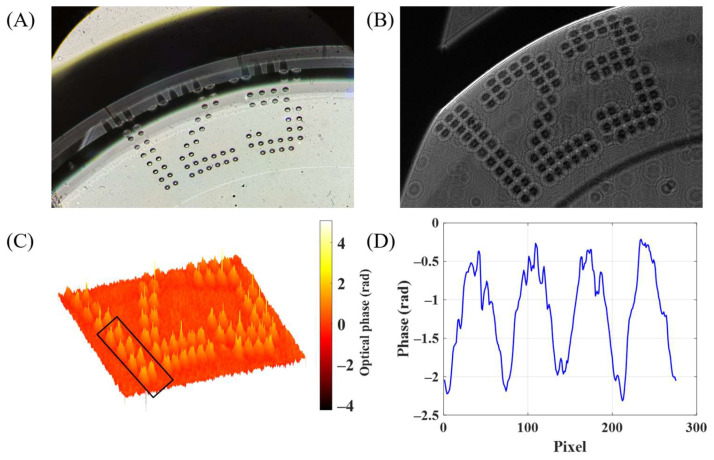
(**A**) Permanent marks of an Acuvue Oasys lens observed with an optical Olympus BX-60 microscope. (**B**) Recorded hologram of the permanent mark. (**C**) Experimentally obtained phase map for this contact lens. (**D**) Experimentally obtained phase profile of four of the mark’s dots marked in the black box in (**C**).

The experimental results of the i-wholoscope to the visualization and profile characterization of engravings in soft contact lenses are presented in Figure 7, Figure 8 and Figure 9, for the three considered contact lenses (Soflens, Miru, and Acuvue Oasys, respectively). The results include the recorded hologram, the retrieved phase distributions after applying full numerical processing, and, in some cases, a phase profile of the inspected marks. In addition, a white light image under a regular upright microscope (Olympus BX-60, Tokyo, Japan) is included just as a qualitative validation of the engraving’s shape. From Figure 7, Figure 8 and Figure 9, we provide local reconstructions because of the defocus introduced by the contact lens surface curvature, since the engravings are at the border of the lenses, preventing them from interfering with the users’ vision.

The defocus effect is especially critical for the engraved dots in the Miru (Figure 8) and Acuvue (Figure 9) lenses, where it becomes notable as the dots fall to the lens border. Finally, the obtained phase profiles from the marks’ parts indicated with black boxes in Figure 7 and Figure 9 show uniform thickness values despite the introduction of irregularities related to their respective engraving techniques.

## 4. Discussion

The aim of this study was to demonstrate the potential effectiveness of the application of the proposed i-wholoscope to the inspection and characterization of soft contact lens engravings. The obtained experimental results provide an optimistic perspective to the application of this technology for the inspection of these marks as well as the inspection of any lens structure that meets the requirements of the Gabor regime, such as surface imperfections or weak diffractive parts of contact lens biosensors [4,5,6,7], where this technique could also be tested.

The inspected lenses show, in general, an absence of polished engravings, and, consequently, there are surface irregularities in the retrieved phase maps. In addition, the results obtained from the Acuvue Oasys lens and Miru’s lens engravings show the need for considering the surface geometry (local curvature) where each of the marks are located. In this preliminary validation, numerical propagation considering plane waves was performed, thus assuming a locally flat surface over the marks placed on a very small area in comparison with the full lens area. Under these circumstances, the inspection of marks needs to be performed in small areas to avoid the effects of introduced aberrations (defocus by sphere, astigmatism, prismatic effect, etc.) related to the curvature of the lenses. Nevertheless, for future applications of this technique, the application of image processing techniques as well as focus stacking ones, or the introduction of a compensating phase-curved term in the propagation calculations or in the illuminating beams, could lead to an artificially increased depth of field as a combination of different focused planes at different distances of the same sample or a single focus of the whole curved surface [51,52,53].

The application of a phase-retrieval algorithm is a widely known and tested tool which has been demonstrated to be very useful in managing the problem of the overlapping of the real and twin-image planes in in-line configurations [44]. This algorithm has played a significant role in the improvement of the quality of our results, since it decreases the twin-image-related noise to the real image plane without compromising the system resolution. Nevertheless, a compromise between twin-image-disturbance reduction and the appearance of a high frequency noise as a consequence of the phase-retrieval algorithm needs to be balanced when choosing the number of iterations. In our case, both aspects are balanced up to N = 20 iterations, since for a greater N, there is no noticeable gain and even high frequency noise contributions start to appear. The coherent noise reduction of the images can also be improved by the introduction of a partially coherent source of light, resulting in better quality results [49].

The main advantage of this simple configuration is the compactness and the cost-effectiveness of it compared with the current commercial microscopy and interferometry techniques. This is a key factor related to the user’s health, comfort, and quality of vision, since the surface of contact lenses is supposed to interact with the tear film. Therefore, its associated parameters, such as the materials, irregularities, or roughness, might affect the stability of this layer and are also correlated with deposit adhesion [54,55,56].

The smaller dimensions of the soft contact lenses and consequently their marks in comparison with the previously demonstrated applications for the permanent engravings in ophthalmic lenses [49,50] require a rigorous calibration of the spatial resolution of the device, whose experimentally obtained values agree with the theoretically expected ones. The resolution limit, mostly limited by the geometry of the sensor, is perfectly matched with the dimensions of the observed engravings, while it can also be improved for smaller marks using a smaller-pixel-size sensor, thus providing results closer to the theoretical diffraction limit. On the other hand, given that the structure of the instrument allows for the introduction of a collimating lens on the illumination, the divergence of the light beam can be varied, obtaining different magnifications on the sensor depending on whether the incident light has a greater divergence (M > 1) or convergence (M < 1) [57,58]. If necessary, this fact would also optimize the sampling resolution criterion. Nonetheless, the resulting field of view (FOV) must be considered depending on the dimensions of the inspected samples. In this case, the available sensor dimensions and the lateral magnification of our configuration provide a good balance between spatial resolution and FOV.

Furthermore, the reduced refractive index step between the immersion medium and the samples needs to be considered to perform an accurate phase measurement calibration, since smaller index differences suppose the obtention of smaller optical path differences and, consequently, smaller phase values for a same geometrical step. The experimental values obtained for the calibrated phase targets agree with the theoretical values, with a similar standard deviation in both cases. Since this calibration provides reliable measurements and the phase data are directly correlated with a constant value with the height and depth of the samples, all the exposed results are available in terms of retrieved phase values, which can be directly translated to thickness units by knowing the exact values of the refractive indexes involved in the process (also by considering their possible surface treatments) at the illumination wavelength. It is worth noting that the exact values of the refractive index of the contact lenses are difficult to obtain, since they can vary depending on different factors, like the water content of each contact lens or the chemical composition of the immersion medium such as a saline solution or different multipurpose conservation solutions commonly used for the maintenance of lenses [59,60,61,62]. Despite this, for general calculations, a refractive index value for saline solutions equal to that of the distilled water can be assumed [63].

## 5. Conclusions

The preliminary experimental results provided by the proposed i-wholoscope constitute an appropriated platform for the inspection (visualization) and characterization (quantitative measurement) of permanent marks present in soft contact lenses. As in PALs’ engravings, contact lens marks exhibit a weak-diffractive behaviour that, in addition with their dimensions and shape, make them good samples to perform Gabor’s holography regime. The calibration process for known phase resolution targets as well as for validation using three commonly used commercially available soft contact lenses are reported here, showing the i-wholoscope as a compact and cost-effective solution for developing new ophthalmic devices aimed at quality control and/or the inspection of the manufactured contact lenses. Nevertheless, the contact lenses’ curvature constitutes an important parameter to consider, whose characterization seems to be a key factor for the holograms’ recording and reconstruction process. Furthermore, the application of iterative algorithms will help to enhance the optical quality of the reconstructed images.

## Data Availability

All data required to reproduce the results can be obtained from the corresponding author upon a reasonable request.
